# Expert consensus on imaging diagnosis and analysis of early correction of childhood malocclusion

**DOI:** 10.1038/s41368-025-00351-1

**Published:** 2025-04-01

**Authors:** Zitong Lin, Chenchen Zhou, Ziyang Hu, Zuyan Zhang, Yong Cheng, Bing Fang, Hong He, Hu Wang, Gang Li, Jun Guo, Weihua Guo, Xiaobing Li, Guangning Zheng, Zhimin Li, Donglin Zeng, Yan Liu, Yuehua Liu, Min Hu, Lunguo Xia, Jihong Zhao, Yaling Song, Huang Li, Jun Ji, Jinlin Song, Lili Chen, Tiemei Wang

**Affiliations:** 1 Department of Oral Radiology, Nanjing Stomatological Hospital, Affiliated Hospital of Medical School, Institute of Stomatology, Nanjing University, Nanjing, China; 2 Department of Pediatric Dentistry, West China Hospital of Stomatology, Sichuan University, Chengdu, China; 3 Department of Oral Radiology, Peking University School and Hospital of Stomatology, Beijing, China; 4 Department of Oral Radiology, School & Hospital of Stomatology, Wuhan University, Wuhan, China; 5 Department of Orthodontics, Shanghai Ninth People’s Hospital, Shanghai Jiao Tong University School of Medicine, Shanghai, China; 6 Department of Orthodontics, School & Hospital of Stomatology, Wuhan University, Wuhan, China; 7 Department of Oral Radiology, West China Hospital of Stomatology, Sichuan University, Chengdu, Sichuan China; 8 State Key Laboratory of Oral & Maxillofacial Reconstruction and Regeneration, National Clinical Research Center for Oral Diseases, Shaanxi Clinical Research Center for Oral Diseases, Department of Oral Radiology, School of Stomatology, Fourth Military Medical University, Xi’an, Shaanxi China; 9 Yunnan Key Laboratory of Stomatology, The Affiliated Hospital of Stomatology, School of Stomatology, Kunming Medical University, Kunming, China; 10 Department of Oral Radiology, School and Hospital of Stomatology, China Medical University, Shenyang, China; 11 Department of Oral and Maxillofacial Radiology, Hospital of Stomatology, Guanghua School of Stomatology, Sun Yat-Sen University, Guangdong Provincial Key Laboratory of Stomatology, Guangzhou, China; 12 Department of Orthodontics, Peking University School and Hospital of Stomatology, Beijing, China; 13 Department of Orthodontics, Shanghai Stomatological Hospital & School of Stomatology, Fudan University, Shanghai, China; 14 Department of Orthodontics, Hospital of Stomatology, Jilin University, Changchun, China; 15 Department of Orthodontics, Shanghai Ninth People’s Hospital, Shanghai Jiao Tong University School of Medicine; College of Stomatology, Shanghai Jiao Tong University, Shanghai, China; 16 Department of Oral and Maxillofacial Surgery, School and Hospital of Stomatology, Wuhan University, Wuhan, China; 17 Department of Geriatric Dentistry, School & Hospital of Stomatology, Wuhan University, Wuhan, China; 18 Department of Orthodontics, Affiliated Hospital of Medical School, Institute of Stomatology, Nanjing University, Nanjing, China; 19 College of Stomatology, Chongqing Medical University, Chongqing Key Laboratory of Oral Diseases, Chongqing Municipal Key Laboratory of Oral Biomedical Engineering of Higher Education, Chongqing, China; 20 Hospital of Stomatology, Guanghua School of Stomatology, Sun Yat-sen University, Guangdong Provincial Key Laboratory of Stomatology, Guangzhou, China

**Keywords:** Malocclusion, Radiography, Paediatric research

## Abstract

Early correction of childhood malocclusion is timely managing morphological, structural, and functional abnormalities at different dentomaxillofacial developmental stages. The selection of appropriate imaging examination and comprehensive radiological diagnosis and analysis play an important role in early correction of childhood malocclusion. This expert consensus is a collaborative effort by multidisciplinary experts in dentistry across the nation based on the current clinical evidence, aiming to provide general guidance on appropriate imaging examination selection, comprehensive and accurate imaging assessment for early orthodontic treatment patients.

## Introduction

Malocclusion is the most common oral disease in children. Early correction of childhood malocclusion is timely managing morphological, structural, and functional abnormalities at different dentomaxillofacial developmental stages. Early treatment aims to eliminate adverse influences of poor oral habits, oral and systemic diseases on dentomaxillofacial development by rational and effective intervention. It also aims to reduce the severity and complexity of malocclusion, achieving more harmonious and aesthetically pleasing dentomaxillofacial morphology, maintaining normal oral function, and improving systemic health.^1,2^

Imaging diagnosis and analysis is crucial in the early correction of childhood malocclusion. The selection of appropriate imaging modalities and comprehensive radiological diagnosis and analysis play an important role in determining the presence of risk factors leading to malocclusion, assessing the necessity for early treatment, and formulating individualized treatment plans. Furthermore, imaging diagnosis and analysis are essential for monitoring treatment process and predicting outcomes. This expert consensus aims to provide guidance on appropriate imaging techniques selecting and comprehensive imaging findings interpreting for childhood malocclusion patients.

## Principles of X-ray Examination in Children

For children with malocclusion, radiological examinations, particularly two-dimensional X-ray imaging, are a crucial tool for pre-treatment assessment. However, due to children’s increased sensitivity to ionizing radiation,^3–5^ understanding and adhering to fundamental principles of radiological examinations is essential. These principles include ALARA, ALADA, and ALADAIP.

- ALARA Principle (As Low As Reasonably Achievable): Since the 1970s, the ALARA principle has been implemented in the field of radiology, emphasizing the importance of dose optimization in X-ray examinations.^6^
- ALADA Principle (As Low As Diagnostically Acceptable): This principle is guided by diagnostic objectives to implement optimal scanning protocols.^7,8^ The ALADA principle emphasizes the actual optimization of radiation doses, not simply minimizing doses.
- ALADAIP Principle (As Low As Diagnostically Acceptable being Indication-oriented and Patient-specific):
  In recent years, cone-beam computed tomography (CBCT) has been widely used in clinic. In dentomaxillofacial radiology, diagnostic imaging is only part of the application of CBCT. While many pathologies may already be diagnosed in 2D, the CBCT is also used needed to determine the surgical strategy or simulate the treatment plan. The wide range of application beyond diagnosis demands more personalized optimization strategies. That is exactly where ALADAIP comes into play. It was introduced to state that the radiation exposure must be As Low As Diagnostically Acceptable being Indication-oriented and Patient-specific. The addition of two letters, I for indication and P for patient, should encourage the clinician to consider personalized optimization.^7,9,10^ This dose optimization focuses on specific exposure needs related to diagnosis, preoperative planning, and treatment planning, as well as whether it involves model creation and/or 3D printing. In short, ALADAIP is a reminder to ask two questions before an x-ray examination: Why exactly is this imaging exam requested? and Who is the patient? The necessity of individualized imaging plans arises from the need to address each patient’s unique clinical presentation and history. Factors such as the presence of specific symptoms, a family history of craniofacial anomalies, and clinical examination findings can warrant earlier or more frequent imaging exam.

For children with malocclusion, radiological examinations should be performed with strict adherence to justification, appropriateness, and dose optimization. Dose should be adjusted based on the patient’s age, growth and gender, and clinical diagnostic purpose (such as reduce the mA of X-ray and the exposure time for younger patients) or use the ‘low-dose’ protocol of the equipment.^4,11,12^ During X-ray examinations, children should be equipped with lead shields to protect radiosensitive organs,^4^ such as thyroid collars, lead aprons for gonadal protection (rectangular or square shields), or upright protective screens with adjustable windows. The lead shields should be no less than 0.5 mmPb, and for mobile lead screens, no less than 2 mmPb. However, it is also essential to ensure that the usage of lead shields does not interfere with imaging of regions of interest.^11^

## Selection of Imaging Modalities for Children with Malocclusion

### Panoramic Radiography and Lateral Cephalometric Radiography

Panoramic radiographs display the entire dentition, as well as the bilateral maxilla and mandible. Although panoramic radiograph presents magnification and distortion sometimes, it remains a routine X-ray examination for pre-orthodontic assessment. For children with malocclusion, panoramic radiography is still the preferred imaging technique for pre-treatment evaluation.^13^ Panoramic radiographs can be used for a comprehensive assessment of deciduous and permanent dentition, including conditions such as tooth caries, periapical periodontitis in deciduous dentition, the development of permanent dentition, dentition crowding or spacing, malformed teeth, supernumerary teeth, congenital tooth absence, and tooth impaction tendency. Additionally, panoramic radiographs can be used to assess whether there is asymmetrical growth of the bilateral maxilla and mandible or some pathological conditions, such as cysts or odontogenic tumors.^13^

Lateral cephalometric radiographs is used to analyze the morphology and structure, the growth and development of dentition and craniofacial bones, and document their changes after orthodontic treatment.^14^ For children requiring early orthodontic intervention, it provides radiological evidence for classifying dento-maxillofacial deformities and assessing asymmetrical growth of mandible and/or maxilla, supporting orthodontic treatment planning, monitoring the treatment process, and evaluating post-treatment outcomes.^15,16^ Moreover, cephalometric imaging has a standardized magnification rate when compared to panoramic radiography. Thus, lateral cephalometric radiography is also a primary X-ray examination method for early orthodontic intervention in children.^17^

### Periapical Radiography

Due to the presence of magnification and distortion in panoramic radiographs, for early orthodontic patients, when the diagnostic information provided by the panoramic radiographs are insufficient, periapical radiographs can be used to further clarify the diagnosis.^18^ Clinical scenarios where periapical radiographs are recommended for further diagnosis includes dental caries, periapical diseases, tooth abnormalities, and tooth fracture.

### CBCT

Although the radiation dose of CBCT has significantly decreased compared to that of spiral CT, it still has a higher radiation dose compared to panoramic radiographs.^19,20^ Given that children are more sensitive to radiation, current international guidelines indicate that the use of CBCT in orthodontic treatment should be based on clinical indications and individualized judgment. CBCT is not routinely recommended for early orthodontic treatment in children.^21,22^ Generally, if a two-dimensional (2D) image is sufficient for diagnosis, a three-dimensional (3D) CBCT may not be necessary. If a CBCT is required, a panoramic or cephalometric image may sometimes be unnecessary. Additionally, if a non-ionizing method can provide the required occlusal model or information, a CBCT may not be warranted. These considerations help minimize patient exposure to radiation.

For children requiring early orthodontic intervention, there must be sufficient justification for using CBCT over conventional radiographic examinations. For early orthodontic patients, CBCT is applicable in the following clinical scenarios:^12^ When pathological or risk factors identified through clinical or two-dimensional imaging require the aid of three-dimensional imaging for treatment planning, it is recommended to weigh the pros and cons and select an appropriate CBCT field of view based on the specific situation. Pathological factors include supernumerary and impacted teeth affecting orthodontic treatment, developmental abnormalities of tooth morphology and structure, concomitant jaw developmental anomalies, and lesions incidentally discovered on two-dimensional imaging. Risk factors assessment includes assessment of temporomandibular joint, upper airway, cleft palate or alveolar ridge defects.^9,23^ And CBCT images should be thoroughly reviewed for incidental findings in areas adjacent to the jaws, such as the paranasal sinuses, skull base, cervical spine, and other relevant structures.

### Magnetic Resonance Imaging (MRI)

MRI, as an imaging technology that can clearly visualize soft tissues without radiation harm to the human body, is primarily used for the evaluation of the temporomandibular joint in early orthodontic patients. MRI can be employed to assess the relationship between the articular disc and the condyle in both temporomandibular joints, the condition of the articular disc, and early changes in the condylar bone.^24^

A summary of advantages, disadvantage, clinical condition and effective dose^11^ of above imaging technique is showed in Table 1. Additionally, it should be emphasized that the effective dose of different imaging technique is quite available according to different equipment and different scanning parameters, especially for CBCT.^19^ The radiation dose of CBCT is significantly influenced by the field of view (FOV), voxel size, and exposure settings (kV and mA). A smaller FOV has smaller radiation dose compared with larger FOV. Increasing voxel accuracy, such as from 400 µm to 200 µm, can double the radiation dose due to the need for more projections. Additionally, variations in kilovoltage (kV) and milliampere (mA) settings across CBCT devices further contribute to dose discrepancies.^9^ So, the radiation dose could be quite variable across different imaging modalities or machines.

**Table 1:** Comparison of different imaging techniques

Imaging Technique	Advantages	Disadvantages	Clinical condition	Effective dose (µSv)	Cost
Panoramic Radiography	Displays entire dentition, maxilla, and mandible	Two-dimensional image Some present magnification and distortion	Pre-treatment and post-treatment for early orthodontic pediatric patients During the treatment process if necessary	14.2–24.3	Medium
Lateral Cephalometric Radiography	Supports treatment planning and monitoring; Provides evidence for deformities classification	Primarily for analysis; not provide detailed information for dentition, maxilla, and mandible	Pre-treatment and post-treatment for early orthodontic pediatric patients During the treatment process if necessary Upper airway assessment Additional bone age assessment	5.6	Medium
Periapical Radiography	Dedicated display for tooth and alveolar bone	Limited regions of interest; Two-dimensional image	Dental caries, periapical diseases, tooth abnormalities, and tooth fracture	1.9–9.5	Low
Cone Beam Computed Tomography (CBCT)	Provides three-dimensional imaging for improved diagnostic accuracy	Higher radiation dose compared to panoramic radiographs	Pathological factors: supernumerary and impacted teeth, developmental abnormalities of tooth morphology and structure, concomitant jaw developmental anomalies, and lesions incidentally discovered on two-dimensional imaging; Risk factors assessment of temporomandibular joint, upper airway, cleft palate or alveolar ridge defects	41.8–94.9	High
Magnetic Resonance Imaging (MRI)	Visualizes soft tissues clearly without radiation exposure	Not typically used for hard tissue assessment; more expensive and less accessible	Temporomandibular joint disk assessment	/	High

### Hand-wrist radiography

Skeletal maturation is a physiological sequence of body changes characterized by phenomenon in which timing could vary among growing subjects due to a different biologic clock.^25^ Determining the skeletal maturation in the treatment planning of early orthodontic patients is important in terms of establishing treatment objectives, the timing of orthopedic treatment, the type of appliance to be used and the duration of the treatment, and predicting treatment outcomes. A child’s developmental stage can be assessed using various parameters, including height, weight, chronological age, secondary sexual characteristics, bone age, and dental age. Among these, bone age is considered the most reliable indicator for evaluating developmental status.^26,27^ The appearance and fusion of different ossification centers follow a distinct pattern and timeline from birth through skeletal maturity. Radiological assessment of skeletal maturity, referred to as bone age, plays a central role in this evaluation.^28^

Hand-wrist radiographs are a commonly used method for assessing bone age due to their simplicity, clarity, and high predictive value. These radiographs provide an estimation of bone age based on the degree of calcification and the morphological changes of specific bones. The wrist region, composed of multiple carpal bones, undergoes development from the appearance of calcification centers to the eventual closure of growth plates, making it a reliable area for evaluating bone age throughout postnatal growth.^29,30^ The Greulich and Pyle atlas remains the most widely used reference for bone age assessment via hand-wrist radiographs.^31^ In China, the China-05 bone age assessment standard has been extensively validated and is widely recommended for clinical use following years of research and expert endorsement.^32^

In recent years, concerns have arisen regarding the necessity of hand-wrist radiographs for bone age assessment in children, primarily due to the additional radiation exposure.^33–37^ Studies have indicated that bone age can also be reliably assessed using cervical vertebrae from lateral cephalometric radiographs. However, researcher caution that when lateral cephalometric radiographs are employed for this purpose, thyroid shield could not be used and thyroid is a radiosensitive organ.^38^ However, current methods for cervical vertebral maturation analysis focus on the second to fourth cervical vertebrae,^39^ and the thyroid gland usually locate below the fourth vertebra,^40^ thus minimizing interference and making cervical vertebral maturation analysis a viable alternative. In rare cases where lead collar interferes cervical vertebrae imaging, a supplementary hand-wrist radiograph may be considered.

**Fig. 1:**
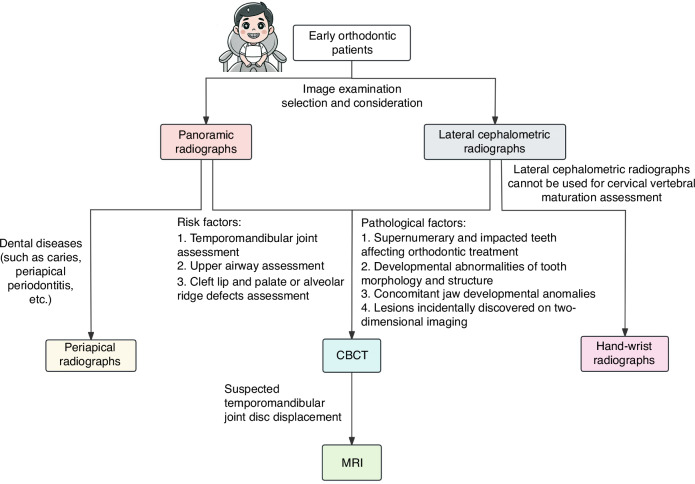
Imaging modality selection for early orthodontic patient

For early orthodontic pediatric patients, since most imaging modalities are X-rays, it is critical to carefully select the most appropriate imaging modality based on clinical scenarios and characteristics of each imaging modality (Fig. 1).

## Radiographic Examination Timing and Frequency and Public Health Recommendations for Early Orthodontic Patients

Early screening and routine radiographic examinations are crucial components of early orthodontic evaluations. These examinations play a vital role in identifying hidden or subclinical conditions that may not be apparent through clinical examination alone. Even when patients present with seemingly normal dentition during initial oral examinations (e.g., during normal tooth replacement), potential issues such as supernumerary teeth, congenitally missing teeth, or impacted teeth may exist without obvious clinical manifestations. Therefore, appropriate radiographic examinations are recommended for these patients, even in the absence of apparent oral problems.

Considering various age groups, risk factors, and clinical presentations, we propose the following radiographic examination schedule:1) Age 6–7: Initial panoramic radiograph to assess dental development and identify potential issues such as supernumerary or missing teeth. 2) Age 8–9: If clinical examination suggests early intervention is necessary, a lateral cephalometric radiograph is recommended to evaluate craniofacial development.^41^ 3) Age 11–12: Second panoramic and lateral cephalometric radiographs to assess permanent dentition eruption and craniofacial development. 4) Age 13–15: If orthodontic treatment is planned, a comprehensive radiographic examination is recommended prior to treatment, including panoramic and lateral cephalometric radiographs, and CBCT if necessary.^9^

From a public health perspective, we recommend:1) Before age 6–7:^42,43^ A panoramic radiographic examination to facilitate early detection of potential dental developmental issues. 2) Age 8-9: A lateral cephalometric radiograph is recommended to evaluate the upper airway and detect problems such as adenoid hypertrophy early.^44^ 3) Age 11–12: A follow-up panoramic radiograph is advised to assess permanent dentition eruption.

To balance effective monitoring with radiation safety during orthodontic treatment, we suggest:^41^1) Pre-treatment: Conduct a comprehensive baseline examination, including panoramic and lateral cephalometric radiographs. 2) During treatment: generally, perform panoramic radiographs every 12–18 months and lateral cephalometric radiographs every 18–24 months. 3) Special circumstances: Increase examination frequency if clinical anomalies are detected or treatment progress is unsatisfactory. 4) Post-treatment: Recommend panoramic and lateral cephalometric radiographs 3-6 months after treatment completion to evaluate treatment outcomes and retention.

Moreover, education is recommended for both clinicians and patients that outline the potential benefits and limitations of radiographic examinations in orthodontics. This could help manage patient expectations and improve compliance with recommended imaging protocols.

## Imaging Assessment and Analysis of Early Orthodontic Patients

### Supernumerary Teeth and Impacted Teeth

**Fig. 2:**
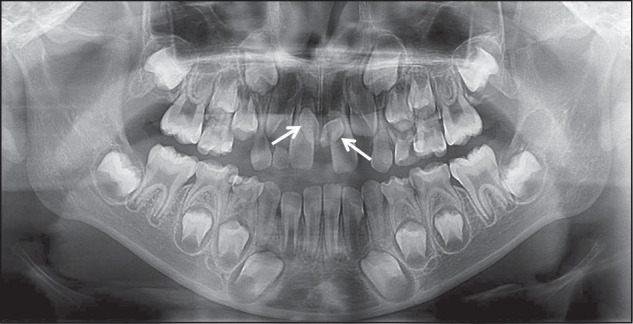
Two supernumerary teeth locate in 11 and 21 regions
- The morphology of supernumerary teeth: teeth with severe root curvature (Fig. 3a) or malformed crown (Fig. 3b).

For early orthodontic patients with supernumerary teeth, the imaging assessment of supernumerary teeth includes the number, the location, the morphology of supernumerary teeth, and the relationship between the supernumerary teeth with the adjacent anatomical structures. Specifically, the imaging assessment includes:

- Confirming the presence of supernumerary tooth and the number of supernumerary teeth (Fig. 2).

**Fig. 3:**
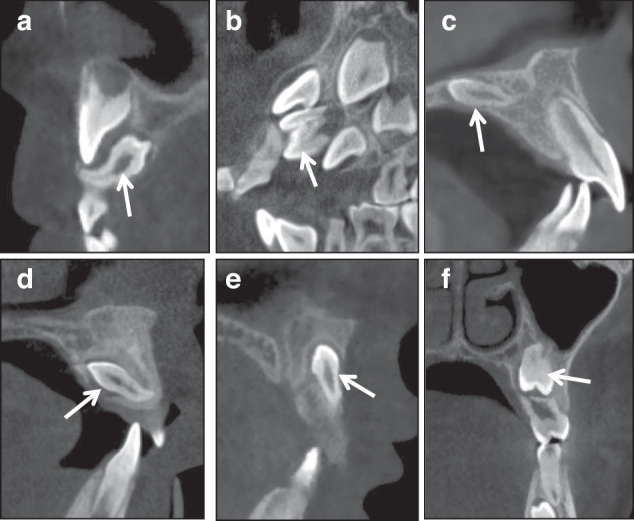
Imaging assessment of supernumerary teeth: (**a**): supernumerary tooth with curved root; (**b**): supernumerary tooth with malformed crown; (**c**): embedded supernumerary teeth located in hard palate, which do not affect the eruption of permanent teeth; (**d**): the supernumerary tooth located in the palatal side, (**e**): the supernumerary tooth located in the labial side; (**f**): supernumerary tooth cause 25 root resorption
- The location of supernumerary teeth: this decides whether the teeth need early intervention or not (Fig. 3c) and the operative approach (Fig. 3d, e).
- The relationship between the supernumerary teeth with adjacent teeth, particularly whether it has caused root resorption of adjacent tooth (Fig. 3f).
- The relationship between supernumerary teeth with adjacent anatomical structures, such as the nasopalatine canal (Fig. 4a).

**Fig. 4:**
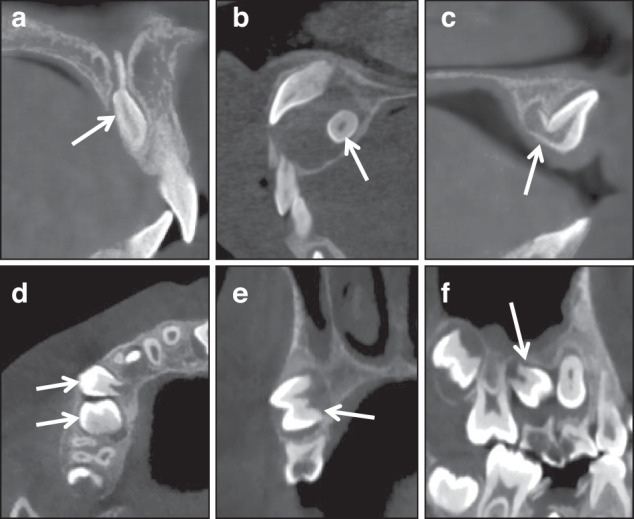
**a** Supernumerary tooth with sharp protuberance in the nasopalatine canal; (**b**): supernumerary teeth accompanied with cyst; (**c**): 11 with curved root and inversion crown and root, which indicates the tooth is an impacted tooth; (**d**–**f**): 14 and 15 show impaction tendency
- Whether there are associated pathological conditions, such as dentigerous cysts (Fig. 4b).

Since the jaw and dentition of early orthodontic patients are still in a dynamic development, and the dento-maxillofacial growth and development vary among different patients, an unerupted tooth is an impacted tooth or not needs to be carefully diagnosed. Some teeth may be diagnosed as impacted teeth (Fig. 4c), while others may only be identified as having impaction tendency (Fig. 4d–f).

### Congenital Missing Teeth

**Fig. 5:**
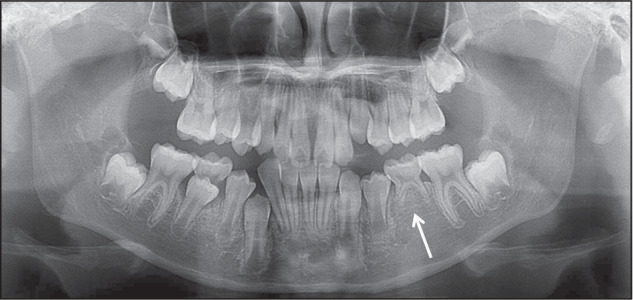
Congenital missing 35

**Fig. 6:**
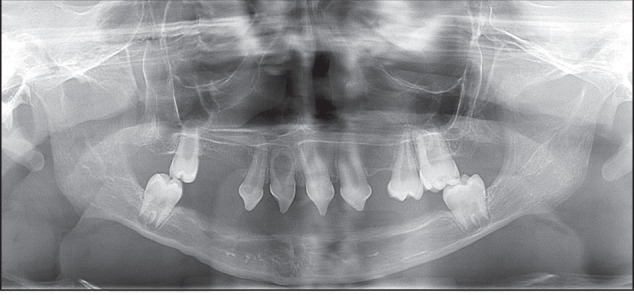
Congenital ectodermal dysplasia

For early orthodontic patients, the imaging assessment of congenital missing teeth (CMT) is also needed^45–48^ (Fig. 5). Patients with congenitally missing permanent teeth may experience a range of complications, including malocclusion, which can lead to functional challenges such as impaired mastication, insufficient alveolar bone development, altered craniofacial relationships, and compromised aesthetics.^49^ Additionally, CMT may be associated with other dental anomalies, such as delayed eruption of other teeth, smaller crown or root size, retention of primary teeth, and abnormal tooth morphology, including taurodontism or peg-shaped maxillary lateral incisors. In cases where multiple permanent teeth are missing, it is imperative to investigate the potential presence of congenital ectodermal dysplasia (Fig. 6).

Imaging modalities play a pivotal role in the accurate diagnosis of CMT.^47,50^ However, since radiographic evidence of tooth germs needs certain level of calcification to appear, inclusion of too young individuals might enter insufficiently calcified tooth buds into the sample, which can be mistakenly diagnosed as missing teeth on the radiograph.^51^ It should be noted that even the initiation of calcification does not guarantee well detection in radiographs; and older ages might be needed for some cases, in order to make sure calcification has reached a detectable minimum.

### Temporomandibular Joint (TMJ)

TMJ plays a critical role in the development of mandible and establishment of occlusion. Radiographic assessment of the TMJ typically focuses on the following key aspects:^52–54^

**Fig. 7:**
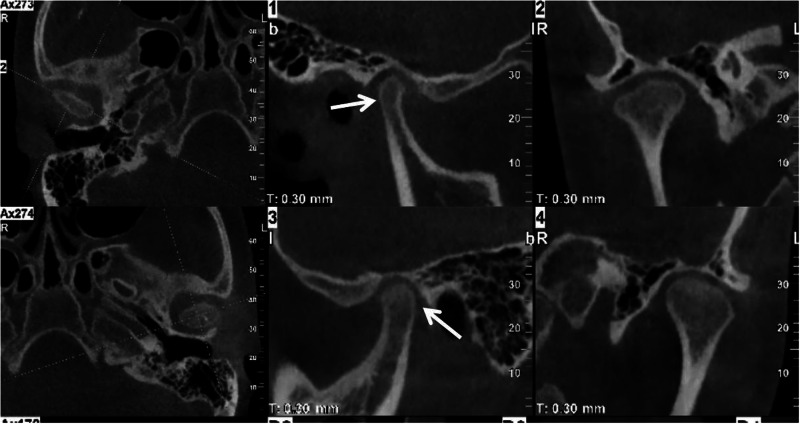
Asymmetry of the bilateral condyles
- Presence of bony changes of the condyles (Fig. 8).

- Symmetry of the bilateral condyles and ascending ramus (Fig. 7).

**Fig. 8:**
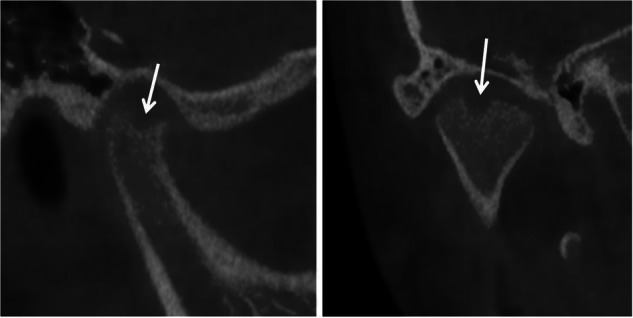
Right condyle with bone resorption
- Presence of reducible or non-reducible displacement of the TMJ disc^55^ (Fig. 9).

**Fig. 9:**
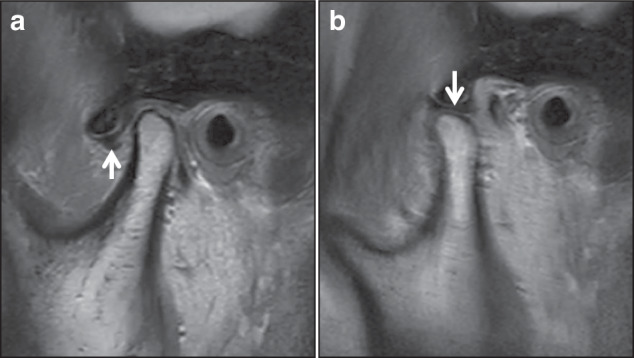
MRI PDWI imaging of anterior disc displacement with reduction, (**a**): in closed mouth position, (**b**): in open mouth position

The first two assessments rely on radiographic imaging, including CBCT, while the evaluation of the disc displacement requires MRI. In cases of condylar asymmetry, a thorough history of trauma should be explored. For pediatric patients, it is important to recognize that the condyles are not fully developed, with their surfaces lacking cortical bone and covered only by a thin layer of calcified tissue. Gradual cortical bone formation is observed after the age of 15. As a result, the cortical outlines of the condyles in children may appear indistinct on radiographs, which should not be misinterpreted as pathological changes.^11^

For early orthodontic pediatric patients, idiopathic condylar resorption (ICR) must be pay attention to. Unilateral ICR may present with mandibular midline deviation and facial asymmetry, while bilateral ICR often manifests as Class II malocclusion, posterior crossbite, premature posterior contact, and varying degrees of anterior open bite. Patients with ICR may also present with symptoms of temporomandibular joint disorders (TMD), either with or without additional clinical signs.^24^ Cephalometric radiographs may show clockwise mandibular rotation, shortening of the posterior facial height, and increased anterior facial height, all contributing to a Class II malocclusion or skeletal discrepancy, often accompanied by increased anterior open bite and overjet. Serial superimposed cephalometric radiographs are valuable for determining whether ICR is in a progressive stage. Panoramic and anteroposterior radiographs typically show reduced condylar volume, flattening of the anterior slope or apex of the condyle, asymmetry of the ascending ramus, and decreased height of the affected ramus. Imaging plays a crucial role in diagnosing condylar resorption in ICR patients.

MRI can provides detailed information about the disc-condyle relationship, the condition of the articular disc, and early indications of changes of the condylar bone.^24^ Given that abnormal disc positioning is a significant contributor to excessive pressure on the condylar bone and subsequent resorption, MRI evaluation of anterior disc displacement is essential in the comprehensive assessment of ICR. Early bone change of ICR in MRI typically reveals discontinuities of the cortical bone, along with multiple resorption pits and irregular, pitted edges.

### Imaging Measurement and Diagnosis of Adenoid Hypertrophy

The upper airway is an anatomical structure involved in essential physiological functions such as respiration, phonation, and swallowing. The craniofacial skeleton provides the structural foundation for the upper airway. Malocclusion can result in alterations in the upper airway structure, while abnormalities of upper airway (such as adenoid hypertrophy) can contribute to the development of malocclusion. Thus, imaging assessment of upper airway is necessary for early orthodontic patients, with particular emphasis on adenoids.

The adenoid is a conglomerate of lymphatic tissue in the posterior nasopharyngeal airway, is part of the pharyngeal lymphoid ring (Waldeyer’s ring). During the growth and development of children, the adenoid can undergo physiological hypertrophy, with its volume typically increasing most rapidly between ages 4–6, peaking around 5–6 years, and gradually regressing after age 10.^44,56^ It is currently believed that adenoid hypertrophy may induce clockwise mandibular rotation, chin underdevelopment, and excessive vertical facial growth, leading to Class II malocclusion. Recent studies have also indicated that these patients may develop Class III malocclusion due to maxillary underdevelopment.^57^ The evaluation of upper airway can often be conducted directly using the patient’s lateral cephalometric radiographs.^14^

The A/N ratio method is the most commonly used approach for assessing adenoid hypertrophy on lateral cephalometric radiographs. Here, “A” refers to the maximum thickness of the adenoid, and “N” is the width of the nasopharyngeal cavity at the most protruding part of the adenoid. By measuring and calculating the *A*/*N* ratio, it is possible to determine the presence of adenoid hypertrophy.^58^ The method for measuring the “*A*” value is relatively standardized in clinical practice, typically referring to the vertical distance between the most convex point of the lower border of the adenoid and the tangent to the external surface of the occipital slope. There are currently multiple methods for measuring the “*N*” value, primarily differing in the selection of reference points.

**Fig. 10:**
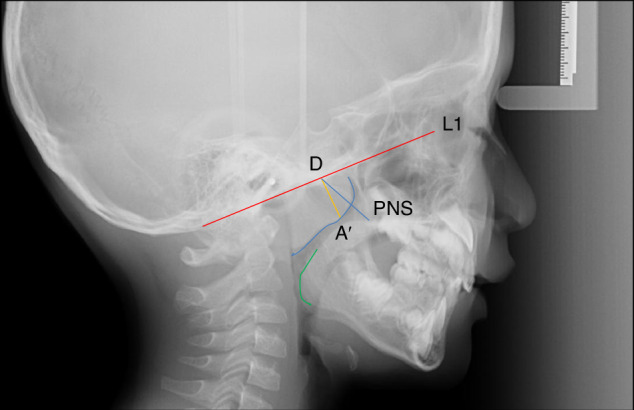
*A*/*N* ratio measurement method of adenoid hypertrophy. L1 represents the tangent line along the external surface of the occipital slope; the blue curve indicates the lower border of the adenoid; point A’ is the most convex point on the lower border of the adenoid; the yellow line segment represents the adenoid width, which is the perpendicular distance from point A’ to line L1 (with point D being the foot of the perpendicular). Point PNS is the posterior nasal spine. The nasopharyngeal airway width (*N*) is the distance between points D and PNS. The green line segment represents the palatine tonsil

The most widely used *A*/*N* ratio analysis method internationally was proposed by Fujioka, an American scholar.^44^ The *N* value is defined as the distance between the posterior superior point of the hard palate to the anteroinferior edge of the spheno-basioccipital synchondrosis. When the synchondrosis is not clearly visualized, the point of crossing of the posteroinferior margin of the lateral pterygoid plate and the floor of the bony nasopharynx was used. The *A*/*N* ratio is obtained by dividing the measurement for *A* by the value for *N*. However, this method has several limitations, such as difficulties in locating the measurement points in some patients.^59^ Chinese scholars^60,61^ also proposed a *A*/*N* ratio method, wherein *A* is the same with the Fujioka method, but the measurement of N is more easily (Fig. 10). The evaluation criteria for the *A*/*N* ratio using this measurement method are:^60^ *A*/*N* value ≤ 0.60 indicates normal size; 0.61–0.70 indicates moderate hypertrophy; and *A*/*N* value ≥ 0.71 indicates pathological hypertrophy.

For adenoid hypertrophy patients, it is also important to note that 1) Adenoid hypertrophy can present as either a uniformly enlarged mass or with a wavy appearance; 2) Adenoid hypertrophy may also be accompanied by palatine tonsil hypertrophy (Fig. 10); 3) Not all cases of adenoid hypertrophy in children will lead to malocclusion, nor will all cases result in airway obstruction. Therefore, imaging evaluation and appropriate follow-up observation are useful for understanding the changes in adenoid hypertrophy over time and for formulating a reasonable treatment plan.

### Craniofacial Congenital Deformities: Alveolar Cleft, Cleidocranial Dysplasia, and Fibrous Dysplasia

Since some patients with congenital craniofacial deformities may seek early orthodontic treatment due to malocclusion, it is essential for clinicians to recognize the radiographic features of these deformities. The diagnosis and comprehensive imaging assessment of these deformities could aid in a more patient-specific treatment planning.

**Fig. 11:**
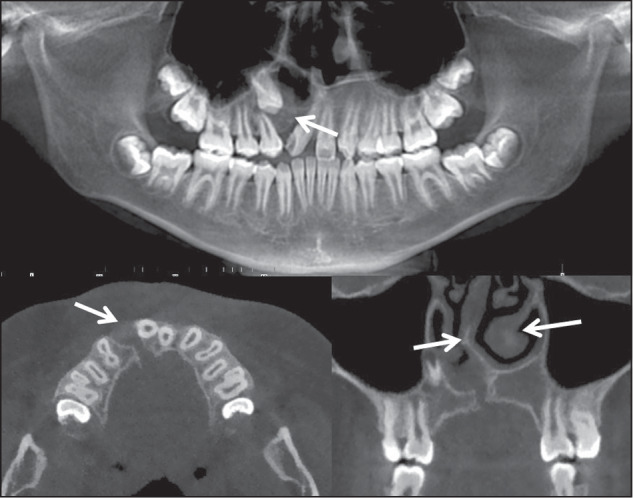
Maxillary alveolar cleft accompanied with impaction of 13, dentition missing of 12 and 22, deviated nasal septum, left inferior turbinate hypertrophy

For alveolar clefts, the imaging assessment should include (Fig. 11):

- The extent and morphology of the alveolar bone defect;
- The presence of missing teeth, impacted teeth, and malformed teeth;
- The presence of deviated nasal septum, turbinate hypertrophy, and maxillary sinusitis.

**Fig. 12:**
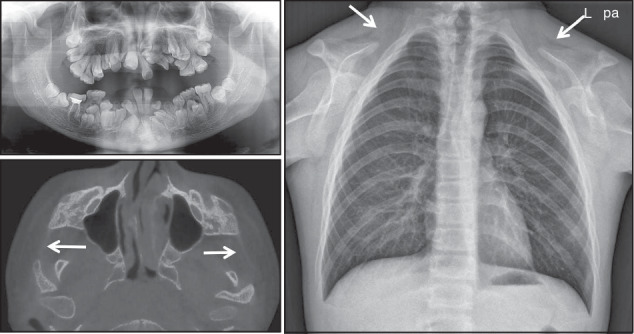
Cleidocranial dysplasia: the panoramic radiograph shows delayed exfoliation of deciduous teeth, retention of permanent teeth, supernumerary teeth, and the CBCT image shows discontinuous zygomatic arch, the chest radiograph shows hypoplastic clavicles

Cleidocranial dysplasia (CCD), is an autosomal dominant skeletal dysplasia characterized by abnormal clavicles, patent sutures and fontanelles, supernumerary teeth, short stature, and a variety of other skeletal changes.^62^ Its radiographic features mainly include (Fig. 12):

- Dental abnormalities: delayed exfoliation of deciduous teeth, retention of permanent teeth, supernumerary teeth, and class III malocclusion.^63^
- Craniofacial abnormalities: inverted pear-shaped calvaria, patency of the anterior fontanelle, midface retrusion and relative mandible prognathism, and some patients with discontinuous zygomatic arch.
- Clavicles: hypoplastic, aplastic, or discontinuous clavicles.^64^
- Spine: hemivertebrae, posterior wedging, spondylolysis and spondylolisthesis.^62,65^

Clinically, if a patient presents with multiple retained primary teeth, delayed eruption of permanent teeth, and numerous impacted supernumerary teeth, an assessment of the above radiographic features is necessary to determine whether the patient is a potential CCD patient.

**Fig. 13:**
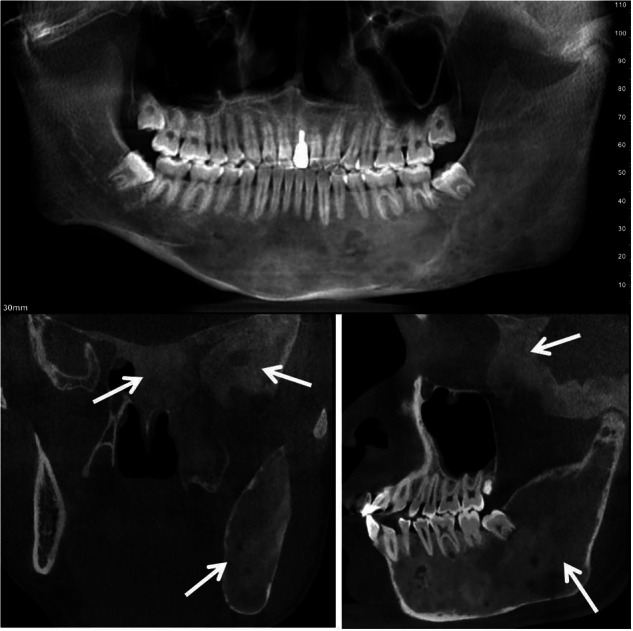
Fibrous dysplasia in mandible, sphenoid bone, temporal bone, the bones present with ground-glass appearance

Fibrous dysplasia (FD) is a non-neoplastic, developmental skeletal disorder. FD may occur in isolation or in association with Café-au-lait pigmented skin lesions, and hyperfunctioning endocrinopathies, termed the McCune-Albright syndrome (MAS).^66^ FD lesions typically manifest during the first few years of life, and expand during childhood and adolescence. Clinically significant bone lesions are usually apparent by age 5 years, with almost no significant lesions appearing after age 15.^67,68^ FD is one type of benign fibro-osseous lesions; the histologic presentation is overlapped with other fibro-osseous lesions which presented with hyperproliferative fibrous material admixed with bony structures, and some elements of woven (irregular) bone. Conservative techniques are recommended, particularly in the pediatric population. However, FD sometimes presents characteristic radiographic appearance, and some experienced clinicians and radiologists could form a diagnosis based on its radiographic features without the need for biopsy. The radiographic features of FD primarily include (Fig. 13):

- Bone changes: radiolucent changes, radiopaque changes, or a combination of radiolucent and radiopaque changes.^11,69^ The radiographic presentation varies according to the degree of maturation of fibrous material in lesions. The characteristic radiographic appearance include “ground-glass” “smoky” and “cloudy” and ‘peau d’orange’ appearance.^70^
- Expansion of the jawbone, with varying degrees of facial asymmetry.
- Malocclusion and dental crowding or spacing secondary to the alveolar bone expanding.

For early orthodontic patients, if bone changes are observed in the mandible or maxilla, the clinicians should exclude whether it is FD, and it is monostotic or polyostotic. In craniofacial region, FD may involve zygomatic bone, sphenoid bone, temporal bone, occipital bone et al. ^65^ so a fully assessment of bone changes of these craniofacial bones is also needed (Fig. 13).

## Artificial Intelligence in Early Orthodontic Treatment

In recent years, the rapid advancement of artificial intelligence (AI) technologies has led to the widespread application of AI-based image recognition and measurement in cephalometric radiographs, as well as AI-driven airway analysis using CBCT)images.^71^ More recently, AI-powered predictive models for assessing bone age and dental age have emerged as a significant focus of research.

Bone age assessment is traditionally conducted through hand-wrist radiographs, where ossification centers and epiphyseal characteristics of the metacarpals, phalanges, carpal bones, and distal radius and ulna are evaluated to determine skeletal development and estimate bone age. AI techniques, through the localization, identification, and extraction of key regional features from hand-wrist radiographs, have proven effective in predicting bone age. Moreover, numerous studies have explored AI technique in predicting cervical vertebral mutation using cephalometric radiographs.^72,73^ These studies consistently demonstrate that artificial neural networks (ANNs) outperform other AI algorithms—such as k-nearest neighbors, naive Bayes, decision trees, and support vector machines—in terms of stability and accuracy, showing high concordance with expert evaluations.^39,74–81^

Dental age estimation, on the other hand, relies on the developmental and calcification stages of dental germs and their eruption sequence in the oral cavity.^82–84^ Conventional methods for dental age assessment include the use of atlases and graded scoring systems. AI-based methods, particularly those employing deep learning, have streamlined this process into three main steps: identification of tooth position, evaluation of tooth developmental stages, and conversion of these developmental stages into dental age. AI has significantly enhanced the efficiency, accuracy, and reproducibility of dental age assessments. However, the development of a comprehensive AI model for dental age estimation that is applicable across diverse populations and age groups remains a challenge and requires further refinement.^85,86^

Beyond these applications, AI has shown promise in assessing the upper airway, a critical aspect of orthodontic treatment planning, particularly for conditions like adenoid hypertrophy that impact craniofacial development. CNN-based models have been used to automate the evaluation of upper airway obstructions.^87^ While these advancements show the potential of AI to enhance diagnostic workflows, it is essential to note that AI systems currently require human supervision to ensure accuracy and alignment with patient-specific needs. Nevertheless, these innovations underline the transformative role of AI in orthodontic imaging diagnostics, paving the way for more efficient and precise patient care.^88^

## Children Anxiety Management

Effective psychological support is crucial for children requiring frequent X-rays, as they may experience varying levels of procedural anxiety due to the unfamiliar environment or discomfort.^89^ Children may feel apprehensive due to the intimidating appearance of bulky imaging equipment, noisy surroundings, and uncertainty about the procedure itself.^89^ For younger patients, this anxiety may manifest as crying, withdrawal, or even challenging behaviors such as shouting or aggression.^90–92^ Neurodiverse children or those with learning disabilities may experience heightened stress, requiring additional consideration and tailored approaches.^89,92,93^

To address these challenges, a child-focused and empathetic approach is crucial. Clear, age-appropriate communication plays a key role in helping children understand the purpose of the examination and what to expect.^89^ Healthcare professionals should use simple language, avoid medical jargon, and engage directly with the child to build trust. Non-verbal communication, such as maintaining eye contact, crouching to the child’s level, and using open body language, further contributes to creating a sense of security.^89,94^

By combining effective communication, empathy, playful distractions, and a supportive environment, healthcare providers can transform the radiological experience for children. These strategies not only alleviate emotional distress but also enhance compliance and improve the quality of diagnostic results, making the process more positive for both children and their cares.^89,90,95^

## Conclusions

Imaging examinations are a critical component of preoperative assessment for early orthodontic patients. Currently, X-ray techniques, including panoramic radiography, cephalometric radiography, periapical radiography, and CBCT, remain the primary imaging techniques. Generally, panoramic radiograph is more suggested for early screening, lateral cephalometric radiograph is recommended for further analysis for early orthodontic treatment, and CBCT is used if necessary. It should be emphasized that the most appropriate imaging examination should be selected based on the diagnostic and therapeutic needs of the patient, adhering to the ALADAIP principle. For early orthodontic patients, in addition to routine imaging assessments, we also need to pay attention to the temporomandibular joint, upper airway, and any potential craniofacial congenital anomalies. A comprehensive imaging assessment will aid clinicians in formulating a more thorough and effective treatment planning. Furthermore, artificial intelligence technologies are increasingly employed in imaging assessment for early orthodontic patients, particularly in the intelligent assessment of bone age and dental age.
